# CBAM-Enhanced CNN-LSTM with Improved DBSCAN for High-Precision Radar-Based Gesture Recognition

**DOI:** 10.3390/s26061835

**Published:** 2026-03-14

**Authors:** Shiwei Yi, Zhenyu Zhao, Tongning Wu

**Affiliations:** China Academy of Information and Communications Technology, Beijing 100191, China; yi_shiwei01@163.com (S.Y.); xiaoyun7k7k@gmail.com (Z.Z.)

**Keywords:** gesture recognition, millimeter wave radar, CBAM-enhanced CNN-LSTM (CECL), clutter suppression, wavelet threshold algorithm

## Abstract

In recent years, radar-based gesture recognition technology has been widely applied in industrial and daily life scenarios. However, increasingly complex application scenarios have imposed higher demands on the accuracy and robustness of gesture recognition algorithms, and challenges such as clutter interference, inter-gesture similarity, and spatial–temporal feature ambiguity limit recognition performance. To address these challenges, a novel framework named CECL, which incorporates the Convolutional Block Attention Module (CBAM) into a Convolutional Neural Network (CNN) and Long Short-Term Memory (LSTM) architecture, is proposed for high-accuracy radar-based gesture recognition. The CBAM adaptively highlights discriminative spatial regions and suppresses irrelevant background, and the CNN-LSTM network captures temporal dynamics across gesture sequences. During gesture signal processing, the Blackman window is applied to suppress spectral leakage. Additionally, a combination of wavelet thresholding and dynamic energy nulling is employed to effectively suppress clutter and enhance feature representation. Furthermore, an improved Density-Based Spatial Clustering of Applications with Noise (DBSCAN) algorithm further eliminates isolated sparse noise while preserving dense and valid target signal regions. Experimental results demonstrate that the proposed algorithm achieves 98.33% average accuracy in gesture classification, outperforming other baseline models. It exhibits excellent recognition performance across various distances and angles, demonstrating significantly enhanced robustness.

## 1. Introduction

With the widespread adoption of 5G technology and the advancement of integrated communication systems, intelligent sensing and interaction has become a hot topic in current research [1,2,3]. Among these, human gesture recognition (HGR) is one of the most active research areas today. As one of the most natural forms of interpersonal communication, hand gestures have long served as an ideal method for human–machine interaction [4]. Consequently, gesture recognition has garnered significant attention and has found extensive applications across various human–machine interaction fields, including smart homes [5], autonomous driving [6], sign language communication [7], electronic device control [8], as well as gaming and virtual reality [9]. These applications enable users to conveniently control devices through gestures, eliminating the need for unnecessary physical contact.

Over the past decade, numerous sensing technologies have been applied in the field of gesture recognition. Initially, wearable sensors such as accelerometers and gyroscopes [10] were used to digitize hand movements into multi-parameter data for gesture recognition. However, this approach often required users to perform complex operations and was prone to interference, lacking convenience in real-world applications. As a result, subsequent research introduced numerous contactless sensing-based solutions. These approaches leverage red, green, and blue (RGB) color information to enhance gesture detection and recognition capabilities. RGB cameras [11] and other sensor-based gesture recognition technologies relying on visual imagery have been steadily advancing, demonstrating outstanding performance in gesture recognition tasks. By tracking and interpreting target gesture images, these systems can accurately identify corresponding hand commands [12], offering a more immersive experience compared to wearable devices. However, vision-based approaches also pose significant challenges: they are highly dependent on environmental conditions, often failing to function properly under bright sunlight or complete darkness [13], and they are typically limited by the visible range of the sensing equipment [14]. Moreover, when hands move rapidly, higher-pixel-count and faster-frame-rate optical sensors become essential for accurate recognition, dramatically increasing computational resource demands [15]. Additionally, employing visual sensors like cameras may raise serious concerns about privacy breaches [16]. In light of these limitations, millimeter-wave radar-based sensing has emerged as a promising alternative for gesture recognition [17]. While radar may slightly lag behind in terms of recognition accuracy, it excels in critical areas such as enhanced privacy protection, robustness against varying lighting conditions, and its compact, easy-to-integrate design [18]. These advantages not only ensure safety but also make radar-based systems more cost-effective and user-friendly for real-world applications.

Currently, gesture recognition methods based on millimeter-wave radar primarily involve three key steps: data acquisition, feature extraction, and classification. Significant progress has already been made in this area through extensive research efforts. In 2015, Google’s Soli project leveraged a 60 GHz Frequency-Modulated Continuous Wave (FMCW) millimeter-wave radar chip to achieve close-range micro-gesture recognition using an end-to-end convolutional recurrent neural network based on range-Doppler features [19]. Meanwhile, Choi et al. [20] demonstrated the successful implementation of sensor-based gesture recognition for 10 distinct hand gestures by employing Long Short-Term Memory networks (LSTM). Additionally, Xia et al. [21] introduced a moving scatter center model to represent 3D point clouds in a multi-dimensional range-Doppler-angle space. A novel scatter-point detection and tracking algorithm was further proposed, which effectively distinguishes between gestures that are easily confused in the range-Doppler domain and ensures robust performance under diverse environmental conditions. Zhang et al. [22] enhanced gesture recognition accuracy by preprocessing radar signals to extract critical information such as distance, angle, and velocity, and then integrating these multi-dimensional parameters into a convolutional neural network, enabling comprehensive utilization of gesture data. To further address the demands of real-time applications, Sun et al. [23] proposed a signal processing framework built on an edge-computing platform, which encodes a comprehensive hand profile into a feature cube and utilizes a lightweight CNN to reduce computational complexity. More recently, Hamza et al. [24] developed a recognition system for tactile displays for the visually impaired, employing a multi-feature encoder to generate time-map spectrograms and achieving high accuracy by adapting the YOLOv8 model as an image classifier.

Improving feature extraction and classification algorithms can significantly enhance the accuracy of gesture recognition methods. However, in practical applications, numerous factors still present serious challenges to the precision of these algorithms. One major issue is the interference caused by noise and clutter originating from either the target body or the surrounding environment—particularly when gestures are recognized at greater distances. Additionally, individual differences in hand movements, such as wrist rotations or subtle joint motions due to personal habits, can further complicate the accurate classification and identification of gestures. Currently, common clutter suppression techniques in gesture recognition research often rely on various constant false alarm rate (CFAR) algorithms, which detect clutter thresholds to effectively eliminate background noise. Yet, this approach tends to perform poorly in complex scenarios. For instance, Ansari et al. [25] introduced a sequential cancellation batch-processing algorithm capable of handling both static and dynamic clutter elimination. Meanwhile, Li et al. [26] proposed a ground-penetrating radar (GPR) denoising method based on automatic phase inversion correction and kurtosis comparison. Furthermore, Allabolu et al. [27] leveraged the inherent high-pass filtering properties of the exponential moving average (EMA) algorithm to develop a novel technique for suppressing stationary clutter while simultaneously enhancing features associated with lateral gesture movements. Despite these advancements, all of the aforementioned clutter suppression methods still exhibit certain limitations or drawbacks when applied in real-world settings.

Addressing the issues mentioned above, this paper proposes a highly accurate gesture recognition scheme based on millimeter-wave radar, from two key perspectives: clutter suppression and classification modeling. Specifically, the Blackman window is employed to mitigate spectral leakage during gesture signal processing. Additionally, by jointly leveraging wavelet thresholding and a dynamic energy nulling algorithm, the method achieves efficient clutter suppression and data augmentation, significantly enhancing the robustness of the algorithm model in real-world applications. Furthermore, by integrating the Range-Doppler (RD) features of gesture recognition datasets and employing an improved Density-Based Spatial Clustering of Applications with Noise (DBSCAN) clustering algorithm based on weighted Euclidean distance, precise segmentation of gestures is achieved. Furthermore, the paper thoroughly considers the dynamic characteristics of human gestures, along with the inherent strengths of Convolutional Neural Network (CNN), Convolutional Block Attention Module (CBAM), and Long Short-Term Memory (LSTM) architectures, to develop a novel CBAM-enhanced CNN-LSTM (CECL) algorithm model tailored for gesture recognition, ultimately achieving superior accuracy in gesture identification tasks.

## 2. Principle of Millimeter-Wave Radar Detection

During the data acquisition phase, the millimeter-wave radar emits a FMCW signal toward the gesture located at a distance R. Within one frequency-modulation cycle, the FMCW signal transmitted by the radar can be expressed as:

(1) $$s_{T}(t)=A_{T}\cdot \cos[2\pi (f_{c}t+\int_{0}^{t}K\tau d\tau )],$$

where t is the fast-time index for each frequency-modulated cycle; $A_{T}$ is the amplitude of the transmitted FMCW signal; $f_{c}$ is the carrier center frequency; K is the frequency modulation slope; and τ is the moment when the signal is transmitted.

The radar receives the reflected signal as:

(2) $$s_{R}(t)=A_{R}\cdot \cos\left\{2\pi [(f_{c}(t-\Delta t)+\int_{0}^{t}[K(\tau -\Delta t)+\Delta f_{d}]d\tau ]\right\},$$

where $A_{R}$ represents the amplitude of the received FMCW signal; ∆t=2R/c is the signal’s round-trip propagation time between transmission and reception (where c is the speed of light); $K\left(\tau -∆t\right)$ denotes the frequency of the radar signal received at time *τ*; and $∆f_{d}$ is the Doppler frequency shift of the signal.

**Figure 1:**
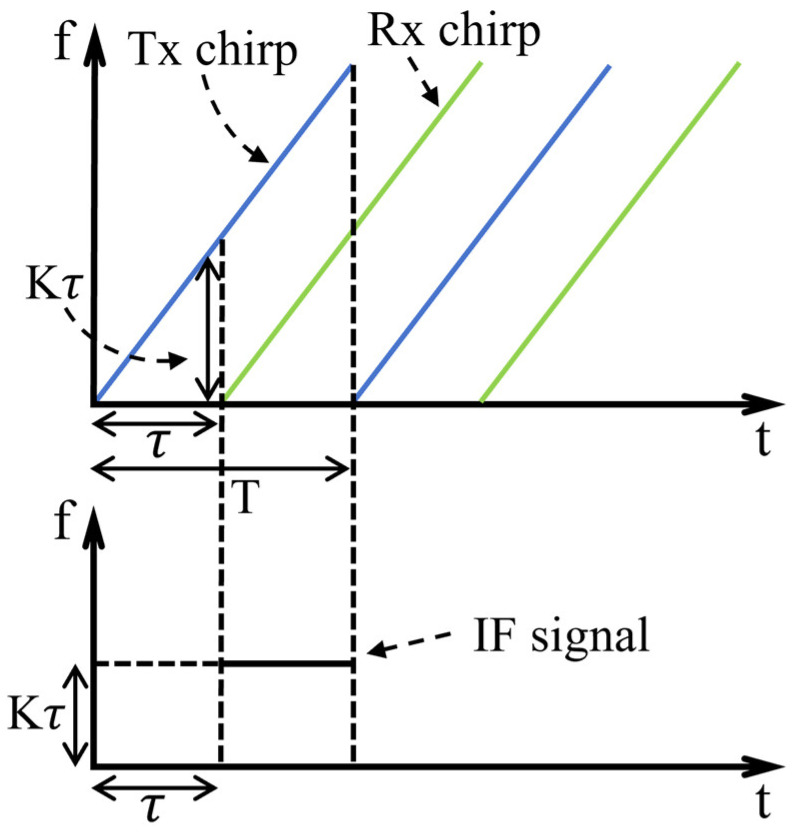
FMCW and IF signals transmitted and received by the radar.

The received waveform and the transmitted waveform are passed through a mixer and a low-pass filter, resulting in an intermediate frequency (IF) signal:

(3) $$s_{IF}(t)=f_{LPF}\left\{s_{T}(t)s_{R}(t)\right\}=A_{T}\cdot A_{R}\cdot \cos\left\{2\pi [f_{c}\Delta t+(f_{IF}-\Delta f_{d})t]\right\},$$

where $f_{IF}=K∆t$ represents the frequency of the IF signal at time t [28]. The process described above is illustrated in Figure 1, where T denotes the sweep period of the chirp signal.

**Figure 2:**
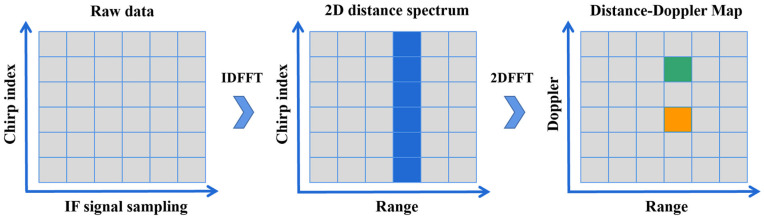
Process for Range Doppler Map (RDM) Generation. The raw data is first processed through IDFFT to obtain the 2D distance spectrum, where the blue column highlights the target range. The final Distance-Doppler map shows two distinct targets at this range, distinguished by green and orange.

Assume that each radar frame contains M chirp signals. For each chirp signal, after reflection and mixing, an IF signal is obtained and sampled at N points. When arranged in rows, these samples form a 2D radar signal matrix of size M×N. For each radar signal matrix, a Fast Fourier Transform (FFT) is applied along the fast-time direction—specifically, the signal sampling direction—resulting in a 2D range spectrum. Next, another FFT is performed on this 2D range spectrum, along the slow-time direction as well as the signal index direction. The outcome is a range-Doppler map (RDM), illustrated in Figure 2. The map is then used as gesture features, which is fed into a classification algorithm model.

The range resolution of the FMCW radar can be derived as follows:

(4) $$R_{res}=\frac{c}{2B},$$

where c is the speed of light, and B is the bandwidth. Therefore, the range resolution of the radar is determined by the bandwidth.

The range measurement in FMCW radar is based on the time delay between the transmitted and received signals. The range of the target (R) can be calculated using the formula:

(5) $$R\;=\;\frac{c\cdot \tau }{2}\;=\;f_{IF}\frac{cT}{2B},$$

where c is the speed of light, τ is the time delay between the transmitted and received signals, $f_{IF}$ is the frequency of the IF signal, and B and T are the bandwidth and the sweep period of the chirp signal. This process can be achieved by FFT for the IF signal.

## 3. Algorithm Design

**Figure 3:**
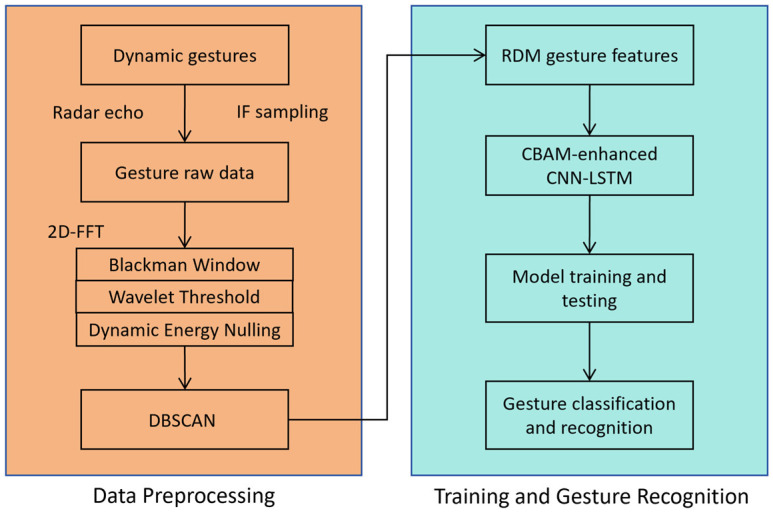
Flowchart of the algorithm.

The workflow of this algorithm is illustrated in Figure 3. First, for dynamic gestures, a millimeter-wave radar is used to collect their echo signals, and the raw gesture data is obtained following IF sampling. Subsequently, the raw data is processed with Two-Dimensional Fast Fourier Transform (2D-FFT).

The resulting range-Doppler maps (RDMs) are sequentially subjected to three preprocessing operations, namely Blackman windowing, wavelet threshold denoising, and dynamic energy nulling, to achieve effective clutter suppression. After that, the DBSCAN algorithm is applied to the processed results to extract RDM gesture features, which are then fed into the training and gesture recognition stage.

In the training and gesture recognition stage, the extracted RDM gesture features are first input into the CECL model integrated with the CBAM for model training and testing. Finally, inference is performed using the trained model to realize gesture classification and recognition.

### 3.1. Noise and Clutter Suppression Algorithm

**Figure 4:**
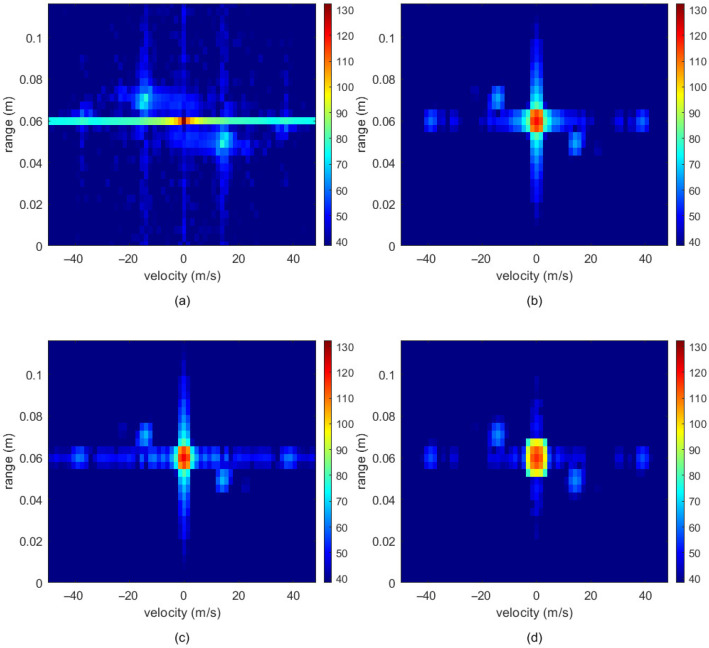
Signal processing effects of different window functions: (**a**) Rectangular window, (**b**) Hanning window, (**c**) Hamming window, (**d**) Blackman window.

**Figure 5:**
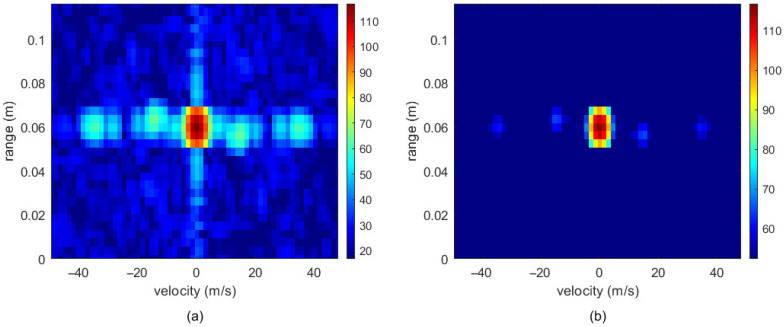
RDM image clutter suppression comparison: (**a**) before clutter suppression and (**b**) after clutter suppression.

**Figure 6:**
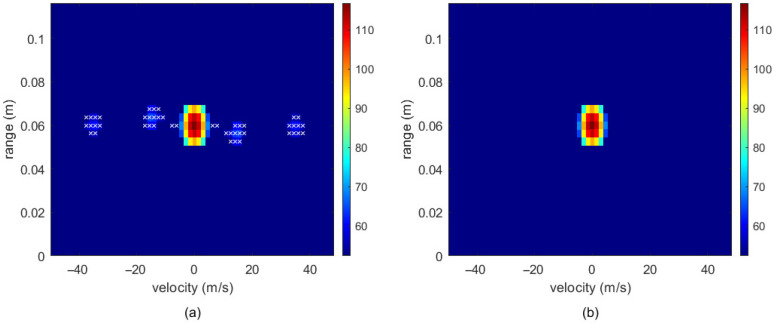
Comparison of RDM processing using DBSCAN: (**a**) before DBSCAN processing and (**b**) after DBSCAN processing. The white “x” indicates the sparse noise points.

In conventional gesture detection methods, processing with 2D-FFT typically results in spectral leakage around the zero-frequency region [29], which can severely disrupt the algorithm model’s ability to detect gesture targets. Therefore, this paper applies a window function after performing the FFT operation. Under identical conditions, the performance of rectangular, Hamming, Hanning, and Blackman windows was compared for handling target points, and the results are shown in Figure 4. Figure 4d illustrates that the Blackman window yielded superior signal-processing performance under the identical experimental conditions. As a representative example, Figure 4, Figure 5 and Figure 6 illustrate the sequential preprocessing results for a single frame of the “swing to the right” gesture after the Range-Doppler transform.

To suppress background noise and enhance the detection performance of moving targets, this paper employs a wavelet thresholding algorithm [30] to reduce background noise clutter. The algorithm utilizes Daubechies wavelets to perform a wavelet decomposition of the RDM matrix, enabling a more accurate estimation in the sense of minimizing the maximum mean squared error asymptotically. The thresholding function is defined as follows:

(6) $$f(x)=\left\{\begin{array}{l} 0,\left|x\right|<\alpha \\ \mathrm{sgn}(x)\left[\left|x\right|-\frac{\alpha^{2}}{\alpha^{4}+e^{\left|x\right|-\alpha }}\right],\left|x\right|\geq \alpha , \end{array}\right.$$

and the threshold α can be expressed as:

(7) $$\alpha =\sigma \sqrt{2\ln N},$$

where σ represents the variance of each FMCW signal. N is the number of sampling points for each IF signal.

Extract the wavelet coefficients $d_{j,k}^{i}$ and the scale factors $c_{j,k}$. Multiply $d_{j,k}^{i}$ by the threshold function (Equation (4)) to obtain a new wavelet coefficient ${d_{j,k}^{i}}^{\prime }$. Then, use $c_{j,k}$ along with this updated wavelet coefficient to reconstruct the RDM spectrum, thereby achieving suppression of background noise and clutter.

Additionally, the motions of different hand joints generate distinct energy and velocity distributions on the RDM. Large-amplitude hand gestures produce substantial radial velocities, whereas joint-centered rotational movements—such as finger flexion or palm rotation—exhibit significant rotational angular velocities. Based on these differences, this paper employs a dynamic nulling strategy [31] to enhance both the positional and velocity information of gestures in the RDM while simultaneously reducing noise interference even further. The dynamic energy nulling strategy can be expressed as:

(8) T(r)=E(r,v=0)−EΔ,

(9) $$E(r,v)=\left\{\begin{array}{l} 0,E(r,v)<T(r)\\ E(r,v),E(r,v)\geq T(r), \end{array}\right.$$

where $E\left(r,v\right)$ represents the value on the RDM corresponding to distance r and velocity v; E∆ is the set threshold.

The above operations effectively enable clutter suppression and enhancement of critical gesture information in the RDM. The results of clutter interference processing in the RDM image are shown in Figure 5.

### 3.2. The Improved DBSCAN Clustering Algorithm Model

DBSCAN is a density-based clustering algorithm that effectively identifies and removes noise points [32]. The DBSCAN algorithm serves as the core algorithm for post-processing noise purification in radar RDMs. After clutter suppression, it further eliminates isolated sparse noise while preserving dense and valid target signal regions, ultimately generating a cleaner RDM for target visualization and analysis. DBSCAN requires no preset number of clusters and can effectively filter out isolated noise points.

For the preprocessed RDM spectrogram, a two-dimensional feature vector [r, d] is assigned to each range-Doppler cell, where r is the distance between the target and radar, and d is the target’s radial Doppler shift. Using these two-dimensional inputs, the density-based spatial clustering algorithm DBSCAN is applied for denoising. The distance metric used in the clustering algorithm is a weighted Euclidean distance that places greater emphasis on the Doppler shift, formulated as follows:

(10) $$Dist(F_{1},F_{2})=\omega_{r}\left|r_{1}-r_{2}\right|+\omega_{d}\left|d_{1}-d_{2}\right|,$$

$\left\{F_{1},F_{2},\ldots ,F_{N}\},\;\;\mathrm{where}\;F_{i}=[r_{i},d_{i}\right]$. The weights are set as $\omega_{r}$ = 0.4 and $\omega_{d}$ = 0.6, as hand gesture motions are primarily reflected in velocity variations. In this study, the neighborhood radius is set to 1.5, and the minimum number of points (MinPts) is configured as 9.

Figure 6 illustrates the distribution changes of noise before and after DBSCAN clustering.

Figure 6a shows the state prior to processing, displaying multiple sparse noise points marked with a white ‘x’ alongside the high-density valid regions. Figure 6b illustrates the result after DBSCAN processing. Relying on its density-based clustering characteristics, these sparse noise points are filtered out, leaving only the original high-density target clusters. It can thus be concluded that DBSCAN possesses the capability to eliminate sparse noise and focus on valid target regions, thereby improving data purity and target distinguishability.

The pseudocode for the DBSCAN procedure is presented in Algorithm 1:
**Algorithm 1.** DBSCAN ProcedureInput: Dataset D, radius Eps, minimum number of points MinPts Output: Clusters C DBSCAN (D, Eps, MinPts) Begin    init C = 0; // initialize the number of clusters to 0    for each unvisited point p in D         mark p as visited; // mark p as visited         N = get Neighbours (p, Eps);         if sizeOf(N) < MinPts then              mark p as Noise; // if sizeOf(N) < MinPts, mark p as noise         else              C = next cluster; // create a new cluster C              ExpandCluster(p, N, C, Eps, MinPts);         end if    end for End The ExpandCluster algorithm pseudocode is as follows: ExpandCluster (p, N, C, Eps, MinPts)    add p to cluster C; // first add the core point to C    for each unvisited point p′ in N         mark p′ as visited;         N′ = getNeighbours(p′, Eps); // perform radius check on all points in N’s neighborhood         if sizeOf(N′) >= MinPts then              N = N + N′; // if greater than MinPts, expand the size of N         end if         if p′ is not member of any cluster              add p′ to cluster C; // add p′ to cluster C         end if    end for End ExpandCluster

### 3.3. The CBAM-Enhanced CNN-LSTM Model

CNN networks (CNNs) and LSTM networks (LSTMs) are two well-established deep learning models, each offering distinct advantages in gesture recognition: CNNs are highly effectively in extracting latent information from input sequences [33,34,35,36], while LSTMs are particularly adept at capturing and distinguishing dynamic temporal patterns [37,38]. In this paper, we leverage the strengths of these two types of neural networks to develop a CNN-LSTM algorithmic model for classifying and recognizing gesture features. This hybrid model combines the complementary benefits of both CNNs and LSTMs—enabling it to not only extract key features from gesture data but also effectively capture the dynamic temporal patterns inherent in such data. As a result, it outperforms traditional classification models such as Support Vector Machines and Random Forests, as well as standalone CNN and LSTM architectures, demonstrating superior prediction accuracy and enhanced robustness against noise and interference [39,40].

To further enhance the model’s ability to focus on salient regions in the RDM, a CBAM is introduced between the CNN and LSTM components. CBAM consists of sequential channel and spatial attention submodules, which adaptively recalibrate feature responses by learning both inter-channel dependencies and spatial significance [41]. Specifically, after the CNN extracts spatial features from each RDM frame, the resulting feature maps are passed through the CBAM, where the spatial attention mechanism highlights motion trajectories with high energy (e.g., hand movement paths) while suppressing noisy or irrelevant areas. This enables the subsequent LSTM to process more discriminative temporal patterns, improving recognition accuracy, especially for gestures with similar velocity patterns but different spatial distributions (e.g., clockwise vs. counterclockwise).

**Figure 7:**
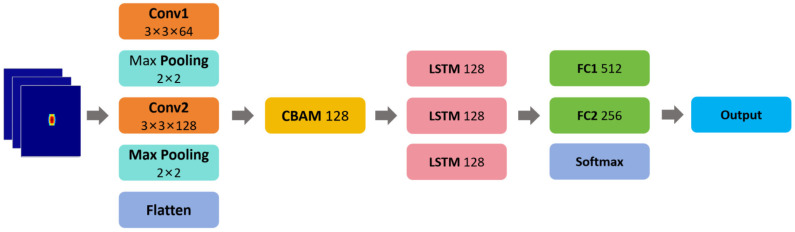
The CECL model structure.

The CECL model architecture proposed in this paper is illustrated in Figure 7. It first uses a CNN to extract features from the RDM of each individual frame of the radar signal, then passes the extracted feature maps through the CBAM, followed by LSTM to capture the evolving patterns across consecutive frames. The CNN module extracts features through a series of convolutional layers, with the number of channels progressively increasing as the network deepens, followed by pooling to reduce the feature dimensions. The resulting feature matrix from the CNN module is then fed into the CBAM, which adaptively refines the spatial and channel-wise feature representations, before being passed to the LSTM neural network structure. Finally, two fully connected layers combined with a Softmax layer are used to perform dimensionality reduction and classification, ultimately yielding probability distribution information for the different gesture categories.

## 4. Experiment Configurations

This experiment employed the IWR6843ISK millimeter-wave radar sensor (Texas Instruments, Dallas, TX, USA) operating in the 60 GHz band, along with a DCA1000 EVM data capture card. The radar module features 4 receiving antennas (Rx) and 3 transmitting antennas (Tx). The radar parameter configuration used in the gesture recognition system is listed in Table 1. The system utilizes a 4 GHz bandwidth, corresponding to a range resolution of approximately 3.75 cm (c/2B), which defines the capability to distinguish closely spaced targets.

To enhance the range precision, zero-padding was employed to extend the FFT length to 512 points, enabling a more precise capture of the target’s spectral peak and achieving a far more accurate estimation of the target’s distance.

**Figure 8:**
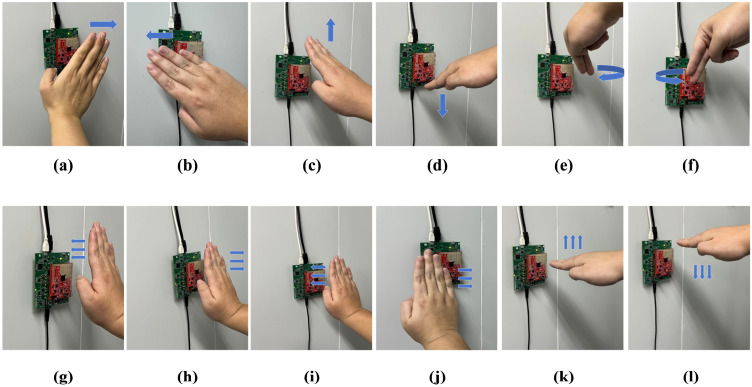
Twelve dynamic gestures: (**a**) swing to the right (**b**) swing to the left (**c**) swing up (**d**) swing down (**e**) swing clockwise (**f**) swing counterclockwise, (**g**) palm forward, (**h**) palm back, (**i**) palm left, (**j**) palm right, (**k**) push upward, and (**l**) push downward.

We recruited 20 volunteers (10 males and 10 females) for the experiment. The dataset comprises 12 distinct dynamic hand gestures, as illustrated in Figure 8. Each participant performed every gesture 30 times. Consequently, the dataset contains a total of 7200 samples (12 gestures × 20 participants × 30 repetitions).

To determine the optimal dataset division strategy, we conducted a comparative analysis of different training–testing splits (6:4, 7:3, 8:2, and 9:1), as shown in Table 2. The results indicated that the 7:3 split provided the best balance between training sufficiency and evaluation reliability. Therefore, the dataset was divided into a training set (5040 samples) and a testing set (2160 samples). The hyperparameters of the proposed model were optimized through experimental tuning, and the final settings were as follows: an initial learning rate of 0.0009, a batch size of 16, 100 training epochs, an exponential decay learning rate schedule, and a decay rate of 0.2.

**Figure 9:**
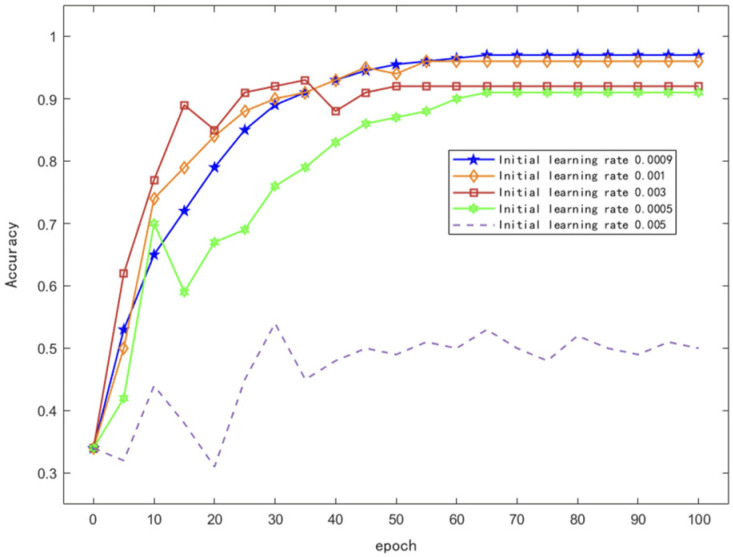
Accuracy of different initial learning rates for various number of epochs.

During the training of the deep learning network, the initial learning rate and decay rate significantly influence the model’s accuracy and loss. Therefore, this study conducted parametric optimization for the initial learning rate in the experiments. The optimization result is shown in Figure 9.

The result illustrates the variation in recognition accuracy of the proposed algorithm under different initial learning rates. It can be observed that when the initial learning rate is either too large or too small, the model’s accuracy exhibits significant fluctuations or fails to converge, and the model fails to reach an optimal performance. The highest recognition accuracy and best convergence performance were achieved when the initial learning rate was set to 0.0009. Therefore, this value was adopted as the initial learning rate in the experiments. Additionally, considering the trade-off between model convergence and computational efficiency, the number of training epochs was set to 100.

## 5. Results and Discussion

The study initially evaluated the system’s real-time recognition performance on six gestures under controlled conditions, with participants maintaining a fixed distance from the radar and keeping their bodies still. Considering that in practical applications the position at which participants perform gestures typically varies, it is crucial to evaluate gesture classification accuracy under diverse spatial conditions. To this end, this study conducts ablation experiments using different models across various distances and angles, aiming to investigate the influence of individual algorithmic components on performance and to validate the effectiveness of the proposed method in improving recognition accuracy.

### 5.1. Real-Time Gesture Recognition Performance

During the data collection process, the participants performed various gestures at a distance of 20 cm in front of the radar and were instructed to keep their bodies still. The confusion matrix for real-time recognition of the six gestures is presented in Table 3. The experimental results indicate that the algorithm exhibits excellent recognition and classification performance for each gesture, achieving an average accuracy of 98.01%.

It is worth noting that distinct kinematic similarities exist between certain gesture pairs, such as opposing directional movements (e.g., swing clockwise vs. swing counterclockwise) or spatially overlapping trajectories (e.g., swing up vs. push upward). Despite this inter-gesture similarity, the confusion matrix reveals minimal misclassification between these categories, validating the model’s discriminative power. This robustness is attributed to the synergistic effect of the proposed method. The DBSCAN clustering filters out body movement noise, ensuring that the input features represent the pure hand trajectory. Furthermore, the CBAM spatial attention focuses on the high-frequency micro-Doppler regions, enhancing the representation of subtle spatial variations. Ultimately, the LSTM layers are instrumental in resolving ambiguities by modeling the unique temporal evolution of each action, allowing the network to distinguish gestures that are spatially similar but temporally distinct.

### 5.2. Impact of Distance

We expanded the testing range to include 50 cm and 100 cm under otherwise identical experimental conditions. This setup allowed for an independent comparison of the base network, attention mechanism, and preprocessing algorithm to assess the framework’s distance robustness. The results are summarized in Table 4, where “Preprocessing” refers to the method detailed earlier.

Comparative experiments between the CNN-LSTM, 3D-CNN, and 3D-ResNet18 architectures under identical preprocessing conditions demonstrate the superiority of the proposed base model. Results indicate that the CNN-LSTM outperforms both 3D convolutional variants across all tests. For instance, at a distance of 20 cm, the CNN-LSTM achieved 97.22% accuracy, surpassing 3D-CNN (94.12%) and 3D-ResNet18 (93.19%). This performance stems from the CNN-LSTM’s ability to decouple spatial feature extraction from temporal modeling. By explicitly modeling long-range sequential dependencies, the LSTM layers effectively capture the complex dynamic evolution of hand movements.

Regarding the attention mechanism, evaluations of the proposed CECL (CBAM-enhanced CNN-LSTM) against CNN-LSTM-SE, CNN-LSTM-ECA, and 3D-CNN-CBAM highlight the benefits of dual-domain attention. While squeeze and excitation (SE) and efficient channel attention (ECA) modules improved the baseline performance to 96.25% and 96.43%, respectively, the CECL model achieved the highest accuracy of 98.01%. Furthermore, the CECL significantly outperformed 3D-CNN-CBAM (95.18%), confirming that the synergy between the CNN-LSTM architecture and CBAM yields superior robustness. This is attributed to CBAM’s mechanism, which refines features along both channel and spatial dimensions, whereas SE and ECA focus exclusively on channel-wise dependencies.

A comparative analysis was then conducted between the proposed preprocessing method (Blackman windowing, wavelet thresholding, dynamic energy nulling, and DBSCAN) and conventional algorithms such as CFAR and OS-CFAR algorithms to validate the preprocessing efficacy. Our method demonstrated distinct advantages in challenging environments, achieving 90.18% accuracy at 100 cm, compared to 89.53% for CFAR and 88.19% for OS-CFAR. This gap implies that standard CFAR methods, relying on amplitude thresholds, struggle to distinguish weak returns from noise at extended ranges. In contrast, our strategy yields higher-fidelity feature maps. Specifically, the density-based DBSCAN effectively identifies cohesive spatial structures, preserving the gesture’s structural integrity and enhancing robustness.

The results show that recognition accuracy decreases as the distance between the target and the radar increases. This degradation is attributed to a combination of physical factors. Primarily, the signal attenuation with distance leads to a continuous decline in the signal-to-noise ratio (SNR) of the acquired signals. Furthermore, the disparity in radar cross-section (RCS) between the hand and the human body becomes more problematic at longer ranges; as the radar beam widens, strong reflections from the torso can overshadow the weaker micro-Doppler signatures of the hand, complicating target isolation. Additionally, multipath effects and environmental clutter become more pronounced relative to the weakened target signal, introducing artifacts that distort the time–frequency features.

Notably, there is no universally accepted industry standard for defining the “operating range”. Generally, an accuracy above 90% is regarded as the threshold for an effective operating range, while an accuracy exceeding 95% is considered a high-precision operating range. Based on our experimental data at a 0° incident angle, we define the system’s effective operating range as 0 to 100 cm. Furthermore, the range of 0 to 50 cm is identified as the high-precision operating zone, satisfying the strict 95% accuracy benchmark.

### 5.3. Impact of Angle

**Figure 10:**
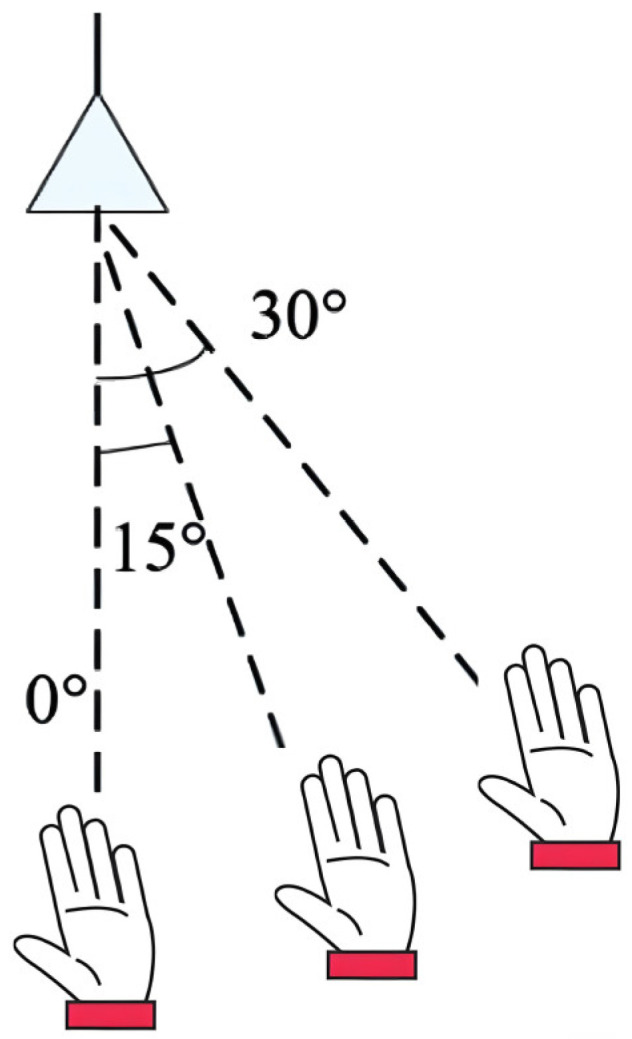
Gesture detection with different incident angles.

Gesture recognition experiments were conducted at various incident angles within a 20 cm range from the radar, as illustrated in Figure 10. For up–down and left–right palm gestures, the incident angle relative to the radar is determined by the central position of the gesture trajectory. The corresponding recognition results are presented in Table 5.

Since Doppler frequency depends on radial velocity, variations in the incident angle significantly affect this critical feature. Moreover, the radar may fail to capture the full trajectory of the hand motion due to angular misalignment, which further distorts the Doppler characteristics of the gesture. As shown in Table 5, angular variation has a considerable impact on the recognition accuracy of the system. Compared with other models, the proposed CECL algorithm exhibits a smaller decline in accuracy under such variations, demonstrating its superior ability to handle feature distortions caused by angle changes.

### 5.4. Robustness Verification

In contrast to the static laboratory setting, we extended the evaluation to a complex scenario containing dynamic interference to simulate real-world background clutter. A robustness test set comprising 240 samples was generated by a newly recruited participant performing 12 gestures (20 repetitions each). During data collection, a distractor walked back and forth at a constant speed of 0.5 m/s, positioned 0.5 m behind the operator (e.g., at 100 cm when the user was at R = 50 cm). The comparative results between the initial test set and the robustness test set are presented in Table 6.

The proposed method demonstrated remarkable robustness to dynamic clutter. For instance, at a distance of 20 cm and a 0° angle, the model maintained an accuracy of 96.87%, showing a decrease of only 1.14% compared to the baseline (98.01%). Even under more challenging conditions (R = 50 cm, Angle = 15°), the accuracy remained robust at 86.08%. These results confirm that the designed preprocessing flow effectively filters out dynamic noise points originating from background movement, ensuring the model focuses on the valid gesture region. The minimal performance drop validates the system’s robustness against individual user differences and environmental interference.

### 5.5. Comparison with State-of-the-Art Methods

We compared the proposed framework with several state-of-the-art radar-based gesture recognition studies published in recent years. Table 7 summarizes the key specifications of these studies.

As illustrated in Table 7, early approaches utilizing traditional machine learning [42,43,44] were generally constrained by limited gesture variety or dataset size, restricting their generalizability in complex scenarios. In the realm of deep learning, while some studies [45,48] report exceptional accuracies, these results are often derived from simpler classification tasks involving fewer gesture categories or smaller datasets. In contrast, our study tackles a significantly more challenging task with 12 distinct dynamic gestures and a large-scale dataset. Despite this increased complexity, the proposed CECL model maintains a leading performance level, surpassing even complex benchmarks such as Transformers and advanced ResNet variants [50,51].

## 6. Conclusions

This paper presents a highly accurate gesture recognition algorithm based on millimeter-wave radar. To address the impact of practical factors—such as distance, angle and clutter—on recognition accuracy, the study focuses on two key aspects: algorithmic modeling and clutter suppression. Specifically, the CECL model specifically tailored for gesture recognition is developed, along with a dynamic energy nulling strategy combined with wavelet thresholding for noise removal and clutter suppression. Furthermore, an enhanced DBSCAN clustering algorithm is employed to eliminate sparse noise and concentrate on relevant regions, significantly improving target discriminability.

Experimental results demonstrate that the proposed algorithm achieves high recognition accuracy, enabling precise real-time gesture detection using a TI IWR6843ISK millimeter-wave radar sensor operating in the 60 GHz band. Furthermore, the study investigates the effects of distance and incident angle on gesture recognition performance. Comparative experiments show that the proposed CECL model and clutter suppression techniques significantly enhance both the accuracy and robustness of the recognition system. Future work will focus on extending the approach to more complex gesture types and real-world application scenarios, as well as exploring multi-radar sensing configurations to further enhance the system’s recognition performance and generalization capability.

## Figures and Tables

**Table 1:** Radar parameter configuration.

Parameters	Value	Parameters	Value
Frequency Range	60~64 GHz	Range Resolution	3.75 cm
Frequency Slope	70 MHz/μs	ADC Sample	256
ADC resolution	12 Bits	ADC Sampling Frequency	4.5 Msps
Number of Frames	1500	Frame Period	40 ms
Number of Chirps	120	Chirp Period	100 μs
TX (Transmit)	3	RX (Receive)	4
TX Power	12 dBm	RX Noise Figure	12 dB
RX Gain	48 dB	RF Gain Target	30 dB

**Table 2:** Performance of Different Dataset Partition Ratios.

Dataset Division Ratio (Training Set:Test Set)	Training Set Size	Test Set Size	Accuracy (%)
6:4	4230	2880	97.43
7:3	5040	2160	98.01
8:2	5760	1440	97.64
9:1	6480	720	97.08

**Table 3:** Classification confusion matrix, (a) swing to the right (b) swing to the left (c) swing up (d) swing down (e) swing clockwise (f) swing counterclockwise, (g) palm forward, (h) palm back, (i) palm left, (j) palm right, (k) push upward, and (l) push downward.

		Real Category											
		(a)	(b)	(c)	(d)	(e)	(f)	(g)	(h)	(i)	(j)	(k)	(l)
Prediction category	(a)	178	0	1	0	0	0	0	0	1	0	1	0
	(b)	1	176	0	1	0	0	2	0	0	0	0	2
	(c)	0	2	177	0	1	0	0	0	0	0	0	0
	(d)	0	0	1	178	0	1	0	0	1	0	2	0
	(e)	0	0	1	0	176	0	1	0	0	1	0	0
	(f)	0	0	0	1	2	177	0	1	1	0	0	1
	(g)	0	2	0	0	0	1	176	0	1	0	0	0
	(h)	0	0	0	0	0	0	1	177	0	1	0	1
	(i)	0	0	0	0	0	0	0	2	175	0	1	0
	(j)	0	0	0	0	1	1	0	0	1	176	0	0
	(k)	0	1	0	0	0	0	0	0	0	2	175	0
	(l)	1	1	0	0	0	0	0	0	0	0	1	176
	Accuracy (%)	98.89	97.78	98.33	98.89	97.78	98.33	97.78	98.33	97.22	97.78	97.22	97.78
	Average accuracy (%)	98.01											

**Table 4:** Average accuracy of gesture recognition at different distances (%).

	R = 20 cm	R = 50 cm	R = 100 cm
Preprocessing + CNN-LSTM	97.22	95.18	89.21
Preprocessing + 3D-CNN	94.12	92.63	84.95
Preprocessing + 3D-ResNet18	93.19	92.03	82.04
Preprocessing + 3D-CNN-CBAM	95.18	93.37	85.27
Preprocessing + CNN-LSTM-SE	96.25	95.46	89.31
Preprocessing + CNN-LSTM-ECA	96.43	95.56	89.40
CFAR + CECL	95.78	93.28	89.53
OS-CFAR + CECL	95.88	93.52	88.19
Preprocessing + CECL	98.01	95.83	90.18

**Table 5:** Average accuracy of gesture recognition at different angles.

	Angle = 0°	Angle = 15°	Angle = 30°
Preprocessing + CNN-LSTM	97.22%	88.56%	75.18%
Preprocessing + 3D-CNN	94.12%	83.06%	68.10%
Preprocessing + 3D-ResNet18	93.19%	84.15%	69.54%
Preprocessing + 3D-CNN-CBAM	95.18%	84.86%	69.12%
Preprocessing + CNN-LSTM-SE	96.25%	87.96%	71.16%
Preprocessing + CNN-LSTM-ECA	96.43%	87.82%	71.25%
CFAR + CECL	95.78%	86.15%	72.03%
OS-CFAR + CECL	95.88%	85.19%	71.67%
Preprocessing + CECL	98.01%	90.04%	80.37%

**Table 6:** Comparison of average accuracy under dynamic clutter interference (robustness test set) and the initial test set.

Distance	Angle	Average Accuracy	
		Robustness Test Set	Initial Test Set
R = 20 cm	Angle = 0°	96.87%	98.01%
	Angle = 15°	88.58%	90.04%
	Angle = 30°	77.58%	80.37%
R = 50 cm	Angle = 0°	93.92%	95.83%
	Angle = 15°	86.08%	88.38%
	Angle = 30°	75.97%	78.15%
R = 100 cm	Angle = 0°	87.58%	90.18%
	Angle = 15°	84.42%	87.04%
	Angle = 30°	73.41%	76.02%

**Table 7:** Performance comparison with existing radar-based gesture recognition methods.

Radar	Range Resolution	Bandwidth	Gestures Number	Dataset Size	Classification Algorithms	Accuracy	Reference
AWR1642 BOOST	3.95 cm	3.8 GHz	6	1250	SVM	98.48%	[42]
Texas Instruments (TI) 77 GHz FMCW	3.75 cm	4 GHz	4	1200	HMM	83.30%	[43]
ST-100 24 GHz K-LC2 Radar	/	/	12	1200	DTW	93.50%	[44]
Infineon Hatvan 60 GHz FMCW	2.50 cm	6 GHz	7	4200	RNN	90.27%	[45]
					CNN	93.58%	
					3D-CNN	99.06%	
AWR1642 BOOST	4.46 cm	3.995 GHz	8	4000	3D-CNN	82.79%	[46]
Infineon BGT60TR24B 60-GHz FMCW	0.60 m	500 MHz	8	1600	CNN-LSTM	94.75%	[47]
TI IWR1443 Evaluation Module	3.75 cm	4 GHz	6	2400	3D-CNN	96.70%	[48]
					CNN-LSTM	99.60%	
Texas Instruments (TI) AWR1642 77 GHz FMCW	3.75 cm	4 GHz	10	4000	CNN	82.77%	[49]
					3D-CNN	88.07%	
					LSTM	90.35%	
					I3D	89.37%	
					I3D + LSTM	93.05%	
Google Soli 60 GHz FMCW	2.50 cm	6 GHz	49	8418	Transformer	93.95%	[50]
AWR1843 BOOST	≥3.75 cm	≤4 GHz	6	2700	VGG-19	93.52%	[51]
					ResNeXt101	93.33%	
					DenseNet161	92.69%	
					S3D	95.37%	[51]
					I3D	94.54%	
					3-D ResNeXt152	95.19%	
					2D/3D-ResNet18 + Deformable + Attention	97.52%	[51]
TI IWR6843ISK	3.75 cm	4 GHz	12	7200	CECL	98.01%	Ours

## Data Availability

The original contributions presented in the study are included in the article, and further inquiries can be directed to the corresponding author.

## Abbreviations

The following abbreviations are used in this manuscript:

HGR	Hand gesture recognition
CNN	Convolutional neural network
CBAM	Convolutional Block Attention Module
CFAR	Constant false alarm rate
OS-CFAR	Order statistic—constant false alarm rate
EMA	Exponential moving average
FMCW	Frequency-Modulated Continuous Wave
FFT	Fast Fourier Transform
RDM	Range-Doppler map
DBSCAN	Density-Based Spatial Clustering of Applications with Noise
LSTM	Long Short-Term Memory
CECL	CBAM-Enhanced CNN-LSTM
GPR	Ground penetrating radar
IF	Intermediate frequency
2D-FFT	Two-Dimensional Fast Fourier Transform
SNR	signal-to-noise ratio
SE	Squeeze and excitation
ECA	Efficient channel attention
RCS	radar cross-section
